# *Candida albicans* Colonizes and Disseminates to the Gastrointestinal Tract in the Presence of the Microbiota in a Severe Combined Immunodeficient Mouse Model

**DOI:** 10.3389/fmicb.2020.619878

**Published:** 2021-01-08

**Authors:** Chien-Hsiung Pan, Hsiu-Jung Lo, Jia-Ying Yan, Yu-Ju Hsiao, Jun-Wei Hsueh, Di-Wei Lin, Tsung-Han Lin, Sze-Hsien Wu, Yee-Chun Chen

**Affiliations:** ^1^National Institute of Infectious Disease and Vaccinology, National Health Research Institutes, Miaoli, Taiwan; ^2^Graduate Institute of Biomedical Sciences, China Medical University, Taichung City, Taiwan; ^3^Graduate Institute of Medicine, Kaohsiung Medical University, Kaohsiung, Taiwan; ^4^Department of Medicine, National Taiwan University, Taipei, Taiwan

**Keywords:** *Candida albicans*, mouse model, gastrointestinal colonization, immunodeficient mice, disseminated candidiasis, interleukin-22

## Abstract

*Candida albican*s is the leading cause of candidemia or other invasive candidiasis. Gastrointestinal colonization has been considered as the primary source of candidemia. However, few established mouse models that mimic this infection route are available. In the present study, we established a mouse model of disseminated candidiasis developed through the translocation of *Candida* from the gut. In this study, we developed a novel *C. albicans* GI colonization and dissemination animal model by using severe combined immunodeficient Rag2^–/–^IL2γc^–/–^ (Rag2γc) mice, which lack functional T, B, NK cells, and IL2γc-dependent signaling. Rag2γc mice were highly susceptible to *C. albicans* gastrointestinal infection even in the presence of the gut microbiota. Within 4 weeks post infection, Rag2γc mice showed dose-dependent weight loss and disseminated candidiasis in more than 58% (7/12) of moribund mice. Histological analysis demonstrated abundant hyphae penetrating the mucosa, with significant neutrophilic infiltration in mice infected with wild-type *C. albicans* but not a filamentation-defective mutant. In moribund Rag2γc mice, the necrotic lesions and disrupted epithelial cells were associated with *C. albicans* hyphae. Notably, removal of the gut microbiota by antibiotics exacerbated the severity of fungal infection in Rag2γc mice, as demonstrated by elevated fungal burdens and accelerated weight loss and death. Furthermore, higher fungal burden and IL-1β expression were prominently noted in the stomach of Rag2γc mice. In fact, a significant increase in circulating proinflammatory cytokines, including IL-6, TNF-α, and IL-10, indicative of a septic response, was evident in infected Rag2γc mice. Additionally, Rag2γc mice exhibited significantly lower levels of IL-22 but not IFN-γ or IL-17A than wild-type B6 mice, suggesting that IL-22 plays a role in *C. albicans* gastrointestinal infection. Collectively, our analysis of the Rag2γc mouse model revealed features of *C. albicans* gastrointestinal colonization and dissemination without the interference from antibiotics or chemotherapeutic agents, thus offering a new investigative tool for delineating the pathogenesis of *C. albicans* and its cross-talk with the gut microbiota.

## Introduction

*Candida* species are the leading fungal pathogens that cause severe healthcare-associated infections in patients who are immunocompromised, critically ill, or have undergone abdominal surgery (Pfaller and Diekema, 2007; Brown et al., 2012; Kullberg and Arendrup, 2015). Overall 30-day mortality in patients with candidemia or other invasive candidiasis remains high (40% or more) even with early initiation of antifungal agents (Kullberg and Arendrup, 2015). *Candida albicans* is the most common cause of invasive candidiasis among various *Candida* species (Pfaller and Diekema, 2007; Kullberg and Arendrup, 2015). *Candida* species represent ubiquitous commensals that constitute part of the normal human skin and gut microbiota (Pfaller and Diekema, 2007; Kullberg and Arendrup, 2015; Pappas et al., 2018). *Candida* colonization is considered as a prerequisite for endogenous infection, and the gut is as an important source for the development of candidemia (Voss et al., 1994; Marco et al., 1999; Chen et al., 2001). However, few established mouse models that mimic this infection route are available.

The current mouse models for *C. albicans* translocation require a combination of gut microbiota removal, neutropenia and mucosal barrier damage (Koh et al., 2008; Bertolini et al., 2019). To mimic an immunocompromised status, animals treated with immunosuppressive agents (Cole et al., 1996) or that have congenital immunodeficiencies in lymphocytes (Cantorna and Balish, 1990; Balish et al., 2001) and/or neutrophils (Jones-Carson et al., 1995) are commonly used. Evidence from studies in immunodeficient mice indicates that defects in T and/or B cells are insufficient to support *C. albicans* translocation (Koh et al., 2008), although oropharyngeal candidiasis is commonly seen in both T-cell-deficient mice (Farah et al., 2002) and in patients with HIV (Li et al., 2013). In addition, neutropenia is another factor associated with mortality in patients with invasive candidiasis (Charles et al., 2003). However, depletion of neutrophils alone was not found to result in fungal dissemination or death in immunocompetent mice (Koh et al., 2008). Considering that *C. albicans* translocation does not always occur even when both the gut microbiota and host immunity have been suppressed to facilitate fungal colonization, chemotherapeutic drugs causing mucosal damage are usually required to induce disseminated candidiasis (Takahashi et al., 2003; Koh et al., 2008; Hirayama et al., 2020). However, translocation of *C. albicans* across epithelial barriers can also occur without iatrogenic or accidental epithelial damage. For example, in severe combined immunodeficiency (SCID) humans lacking functional T, B, and NK cells due to adenosine deaminase (ADA) deficiency or an IL-2 common receptor gamma chain (IL2γc) mutation (atypical X-linked SCID), opportunistic fungal infections are commonly seen in early life (Fischer, 2000; Kumrah et al., 2020). This immunodeficient condition, i.e., T, B, and NK cell depletion, may render the establishment of natural *C. albicans* colonization and translocation in mice.

Herein, we tested the possibility of establishing a *C. albicans* gastrointestinal (GI) colonization and translocation model by using severe combined immunodeficient Rag2^–/–^IL2γc^–/–^ (Rag2γc) mice. These mice harbor genetic defects in both recombinase gene 2 (Rag2) and IL2γc, resulting in deficiencies in T, B, and NK cells, as well as abnormal signaling of IL2γc-dependent cytokines, including IL-2, IL-4, IL-7, IL-9, IL-15, and IL-21 (Colucci et al., 1999), as observed in humans with IL2γc mutant X-linked SCID humans. Rag2γc mice have been shown to be highly susceptible to systemic *C. albicans* infection (Bar et al., 2014). However, their susceptibility to GI infection is unknown. In this study, we investigated the susceptibility of alymphoid Rag2γc mice to *C. albicans* GI infection and subsequent dissemination, as well as the pathogenic features of this important opportunistic fungus.

## Results

### *C. albicans* Colonized the Gastrointestinal Tracts of Rag2γc Mice in a Dose-Dependent Manner

**FIGURE 1:**
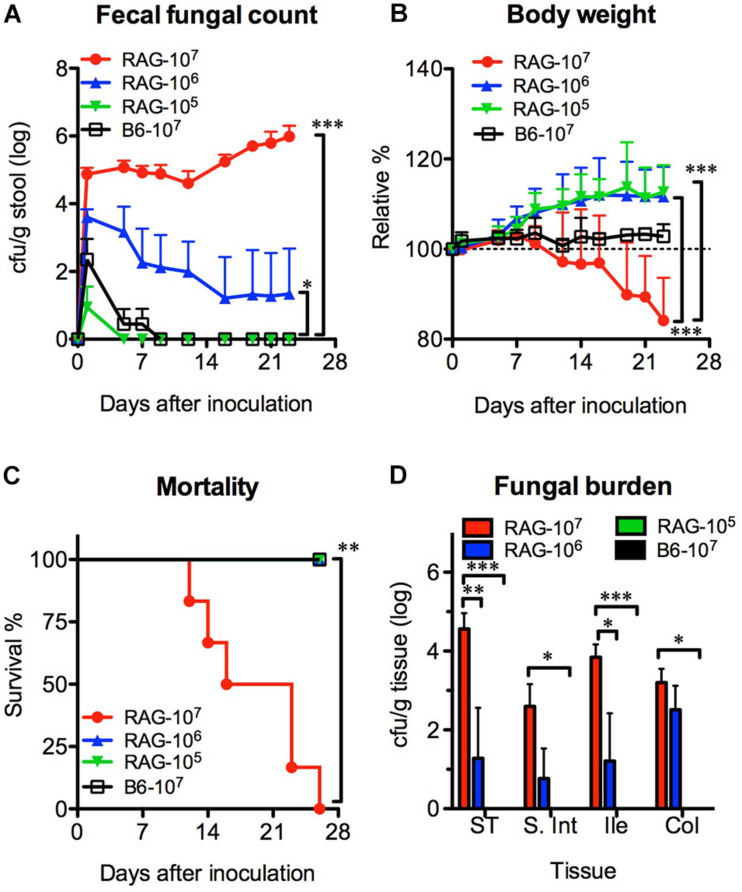
The colonization and dissemination of *C. albicans* in Rag2γc mice was dose-dependent. Groups of Rag2γc (RAG) or B6 mice (*n* = 6) were infected with either 1 × 10^7^, 1 × 10^6^, or 1 × 10^5^ cells of *Candida albicans* strain SC5314 by oral gavage. The fecal fungal counts **(A)**, body weight **(B)**, and the survival rates **(C)** from two independent animal experiments are combined and presented as the means value and SD. **(D)** On day 12, GI tissues from infected mice (*n* = 2), including tissues from the stomach (ST), small intestine (S. Int), ileum (Ile), and colon (Col), were collected and homogenized, and the homogenates were used for cultures. The numbers of colonies were normalized to the tissue weights and presented as the means values with SD. For statistical analysis, 1-way ANOVA **(A,B)** and the Mantel-Cox test **(C)** were used (*, **, and *** for *p* < 0.05, *p* < 0.01, and *p* < 0.001, respectively).

We orally infected Rag2γc mice with 1 × 10^7^ (high dose), 1 × 10^6^ (moderate dose), or 1 × 10^5^ (low dose) *C. albicans* SC5314 cells. B6 mice infected with high-dose SC5314 were used as controls. Without antibiotic treatment, *Candida* colonies were transiently detectable (≤7 days) in the feces of B6 mice and the low-dose group of Rag2γc mice after infection (Figure 1A). In contrast, the *Candida* colonies were persistently detectable in the feces of Rag2γc mice until day 28 or moribundity in the high- and moderate-dose groups; higher fungal colony counts were detected in these groups than in the low-dose group (*p* < 0.001 and *p* < 0.05 by 1-way ANOVA, respectively). Compared to mice in the other two groups, Rag2γc mice in the high-dose group exhibited significant weight loss (*p* < 0.001 by 1-way ANOVA, Figure 1B), and 100% of these mice died within 4 weeks (*p* < 0.01 by the Mantel-Cox test, Figure 1C).

To confirm the localization of *C. albicans* in the GI tract, we sacrificed mice 12 days after inoculation and measured the fungal burdens. Consistent with the fecal fungal counts, *Candida* colonies were detectable in the tissue cultures from the high-dose and moderate-dose groups (Figure 1D); the fungal burden increased in a dose-dependent manner (*p* < 0.01 and *p* < 0.001 for the high-dose group vs. the moderate-dose and low-dose groups, respectively) with the highest fungal burden in the stomach. Although the group of Rag2γc mice infected with moderate dose did not show any death within 4 weeks, we did observe one mouse (6.3%; 1 death among the total of 16 mice in the moderate-dose group in two independent experiments) showed persistent fecal fungal counts and gradual body weight loss until the humane endpoint (>20% body weight loss) was reached 42 days after inoculation.

### Hyphal Formation Was Required for *C. albicans* to Cause Gastrointestinal Candidiasis

**FIGURE 2:**
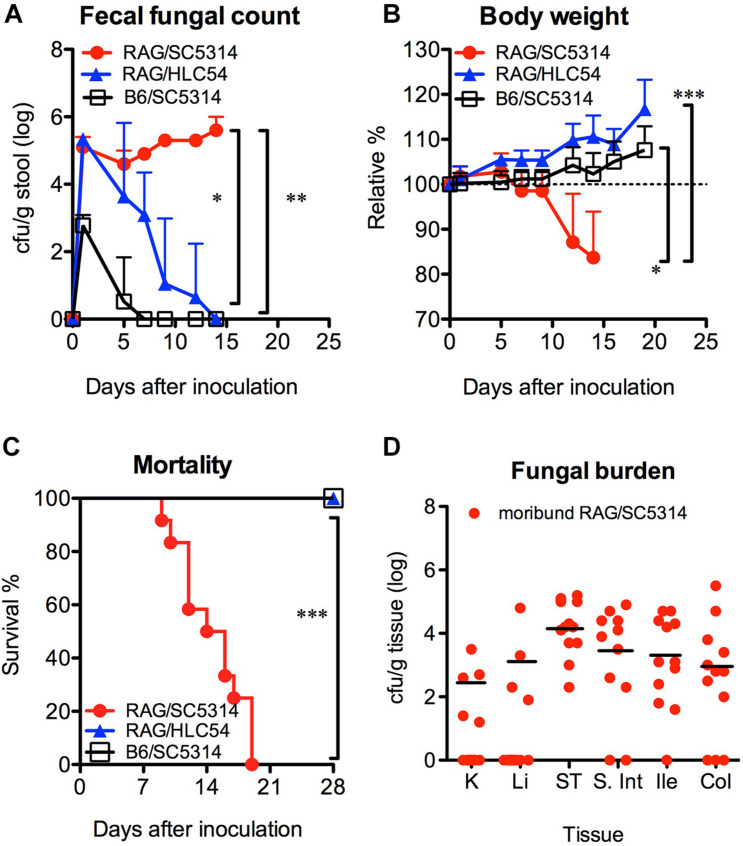
Hyphal formation was required for the colonization and dissemination of *C. albicans* in Rag2γc mice. Rag2γc (RAG; *n* = 12) or B6 (*n* = 8) mice were infected by oral gavage with either the wild-type SC5314 strain or the HLC54 filamentation-defective mutant of *C. albicans*. The fecal fungal counts **(A)**, body weight **(B)**, and survival rates **(C)** from three independent animal experiments are combined and presented as the mean values with SD. SC5314-infected Rag2γc mice were sacrificed when they became moribund. **(D)** Tissues, including the kidneys (K), liver (Li), stomach (ST), small intestine (S. Int), ileum (Ile), and colon (Col) tissues, were collected and homogenized, and the homogenates were used for cultures. The numbers of colonies were normalized to the tissue weights and are presented for each individual mouse (dots) and as the average values for mice with positive cultures (black line). For statistical analysis, 1-way ANOVA **(A,B)** and the Mantel-Cox test **(C)** were used (*, **, and ***, for *p* < 0.05, *p* < 0.01 and *p* < 0.001, respectively).

Formation of hyphae has been reported to be a requirement for *C. albicans* invasion (Lo et al., 1997). Therefore, we compared the susceptibility of Rag2γc mice to GI infection with wild-type SC5314 and the filamentation-defective mutant HLC54. B6 mice infected with SC5314 were used as controls. Fecal fungal counts were detectable in all three groups, but only SC5314-infected Rag2γc mice persistently showed a significantly higher fecal fungal count than mice in the HLC54 and B6 groups (*p* < 0.05 and *p* < 0.01 by 1-way ANOVA, respectively; *n* = 12; Figure 2A). In addition, only Rag2γc mice infected with SC5314 exhibited significant weight loss (*p* < 0.05 and *p* < 0.001 by 1-way ANOVA compared to the HLC54 and B6 groups, respectively; Figure 2B), and 100% of these mice died within 4 weeks (Figure 2C; *p* < 0.001 by the Mantel-Cox test).

To determine the presence of candidiasis, we sacrificed SC5314-infected Rag2γc mice when they became moribund (15–22 days after infection) and healthy HLC54-infected Rag2γc and SC5314-infected B6 mice on day 22. Consistent with the fecal fungal counts, only SC5314-infected Rag2γc mice had *Candida* in the GI tract (data not shown). In addition, seven of 12 (58%) moribund mice had fungal burdens in the liver and/or kidney; the average fungal burdens for the mice showing positive cultures in the kidney and liver were 2.3 and 3.1 log cfu/g tissue, respectively (Figure 2D). These data suggested that disseminated candidiasis developed in SC5314-infected Rag2γc mice without the prerequisite treatments.

### Histological Features Showed That *C. albicans* Disseminated From an Endogenous Origin in Rag2γc Mice

**FIGURE 3:**
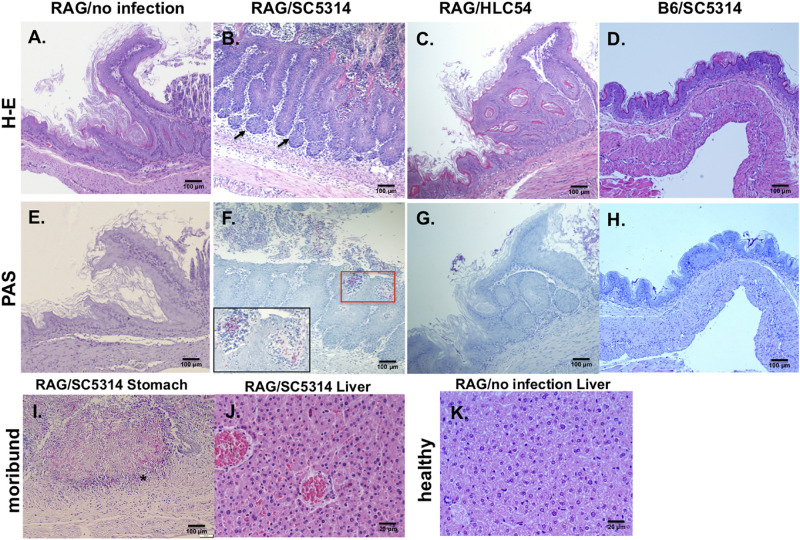
Histological features of wild-type *C. albicans* caused disseminated candidiasis in Rag2γc mice. Rag2γc mice were either uninfected **(A,E,K)** or infected by oral gavage with 1 × 10^7^ wild-type SC5314 **(B,F,I,J)** or HLC54 mutant cells **(C,G)**. B6 mice were infected by oral gavage with 1 × 10^7^ wild-type SC5314 cells **(D,H)** as controls. **(A–H)** Infected mice were sacrificed 12 days post infection and stomach tissues were collected and fixed for H-E **(A–D)** or PAS **(E–H)** staining. The locations in the gastric mucosa showing focal hyperplasia are marked with arrows. A magnified image of the *C. albicans* hyphae located in the red square is shown inside the box in the lower left corner. **(I,J)** The SC5314-infected Rag2γc mice were sacrificed when they became moribund (15–22 days after infection) and tissues were collected for staining. Typical images of stomach tissue stained with PAS **(I)** and H-E stained liver tissues from moribund mice **(J)** or healthy control **(K)** are shown. The location of hyphae penetrated into the muscular layer of the stomach is indicated with an asterisk.

In comparison to the uninfected Rag2γc mice, stomach tissues collected 12 days after infection showed severe tissue lesions, including focal hyperplasia and erosions, in the stomach of SC5314-infected Rag2γc mice but not in B6 mice or HLC54-infected Rag2γc mice (Figures 3A–D). To confirm that this tissue damage was caused by *C. albicans*, we stained the fungi with PAS staining. Consistent with the tissue fungal cultures, *C. albicans* was identified in all SC5314-infected Rag2γc mice but not in B6 mice or HLC54-infected Rag2γc mice, and fungi were located primarily in the squamous-glandular epithelial junction of the stomach (Figures 3E–H). Both the hyphal and yeast forms of *C. albicans* were identified in the damaged mucosa near the gastric pits (Figure 3F, inside the boxed area) and in the disrupted epithelial layer. To confirm disseminated candidiasis, we performed histological examination of moribund SC5314-infected Rag2γc mice. We observed necrotic damage accompanied by abundant hyphae in the muscular layer of the stomach (Figure 3I). In the liver tissue section, the hepatic congestion was noticed in moribund Rag2γc mice (Figure 3J) but not in uninfected controls (Figure 3K).

### Antibiotic Treatment Exacerbated the Severity of Disseminated Candidiasis in Rag2γc Mice

**FIGURE 4:**
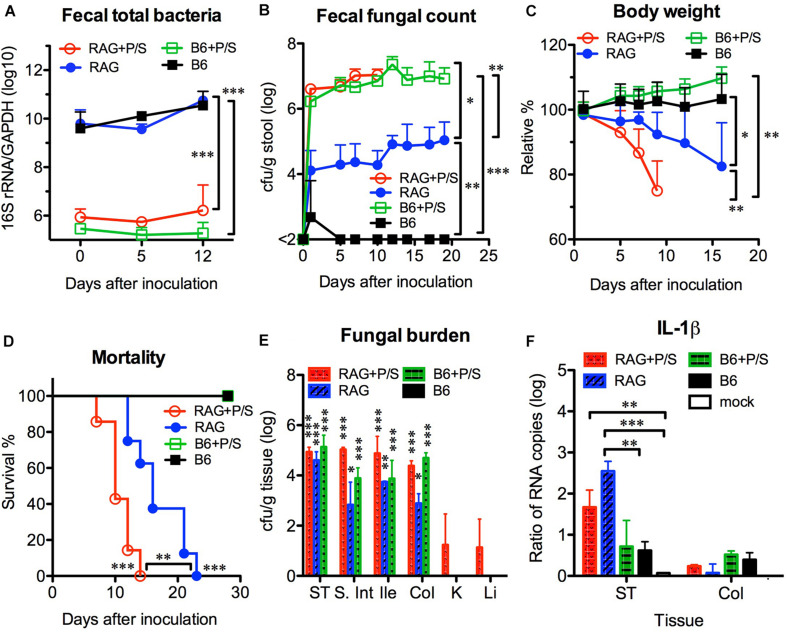
Antibiotic treatment enhanced the severity of *Candida albicans* GI infection in Rag2γc mice. Groups of Rag2γc (RAG; *n* = 8) or B6 (*n* = 6) mice either treated with antibiotics (P/S) or not treated were infected with 1 × 10^7^ *C. albicans* SC5314 cells by oral gavage. The abundance of total fecal bacteria was determined by qPCR of the bacterial 16S rRNA gene **(A)**. The fecal fungal counts **(B)**, body weight changes **(C)**, and survival rates **(D)** from two independent animal experiments are combined and presented as the mean value with SD. Two mice from each group were sacrificed 14 days after infection, and the tissue fungal burden **(E)** and tissue level of IL-1β RNA **(F)** were assayed by culture and qRT-PCR, respectively. For statistical analysis, 1-way ANOVA **(A–C)** and the Mantel-Cox test **(D)** were used (*, **, and ***, for *p* < 0.05, *p* < 0.01 and *p* < 0.001, respectively).

According to the current knowledge, the removal of gut bacteria with antibiotics is able to facilitate *C. albicans* colonization in the gut. To address the influence of the gut microbiota, we orally infected mice with *C. albicans* SC5314 in the presence or absence of antibiotics. After adding penicillin/streptomycin to the drinking water for 5 days, no bacteria were found in stool cultures (in an aerobic condition) from antibiotic-treated mice (data not shown), and the abundance of total fecal bacteria was significantly decreased by over 10,000-fold in antibiotic-treated mice compared with untreated controls (*p* < 0.001, Figure 4A). However, no significant difference was found in the fecal bacterial abundance between the B6 and Rag2γc mice within the same antibiotic-treated or untreated groups. After *C. albicans* GI infection, both groups of antibiotic-treated B6 and Rag2γc mice showed persistently higher fecal fungal counts than untreated Rag2γc mice (>10^6^ vs. ∼10^4^ cfu/g feces; *p* < 0.05 and *p* < 0.01 by 1-way ANOVA for antibiotic-treated Rag2γc and B6 mice vs. untreated Rag2γc mice, respectively; Figure 4B). This suggested that *C. albicans* colonization was enhanced in both Rag2γc and B6 mice treated with antibiotics. However, regardless of antibiotic treatment, only the Rag2γc mice, not the B6 mice, displayed significant weight loss and death within 4 weeks of infection (Figures 4C–D). Compared to the untreated Rag2γc mice, the antibiotic-treated Rag2γc mice had reduced survival times (median survival time of 10 days vs. 16 days; *p* < 0.01) and faster weight loss (mean weight loss of 25% vs. 8% on day 9; *p* < 0.01). This suggested that the depletion of the gut microbiota enhanced the severity of *C. albicans* GI infection.

Despite the high fungal burdens found in the GI tract of antibiotic-treated B6 mice, *C. albicans* did not translocate or cause disseminated candidiasis in these mice. To address the reason for the high susceptibility of Rag2γc mice to *C. albicans* GI infection, we first investigated the localization of *C. albicans* in B6 and Rag2γc mice by harvesting tissues from the GI tract and internal organs 14 days after infection. We found that all groups, except for the untreated B6 mice, had detectable fungal burdens in their GI tissues, which were highest in the stomach (Figure 4E). Although the antibiotic-treated Rag2γc and B6 mice had higher fecal fungal counts than the untreated Rag2γc mice, no significant differences in the stomach fungal burdens were found among the three groups. Moreover, the fungal burden varied across different regions of the GI tract in untreated Rag2γc mice but were maintained at high levels in antibiotic-treated Rag2γc and B6 mice. Because *C. albicans* translocation usually causes local tissue damage and inflammation, we further examined tissue inflammation in B6 and Rag2γc mice by measuring the level of IL-1β expression in the GI tract. Consistent with the survival data, significantly increased levels of IL-1β RNA were detected in the stomachs of Rag2γc mice but not B6 mice compared to those in uninfected mice, irrespective of antibiotic treatment (Figure 4F). In contrast, the level of colonic IL-1β RNA did not differ between the Rag2γc and B6 mice. This finding suggested that the tissue damage caused by *C. albicans* was localized only to the stomach in Rag2γc mice but not in antibiotic-treated B6 mice, even though the latter had higher fungal burdens than the former.

### Gastric Inflammation and Necrotic Damage Occurred in *C. albicans*-Colonized Rag2γc Mice but Not in B6 Mice

**FIGURE 5:**
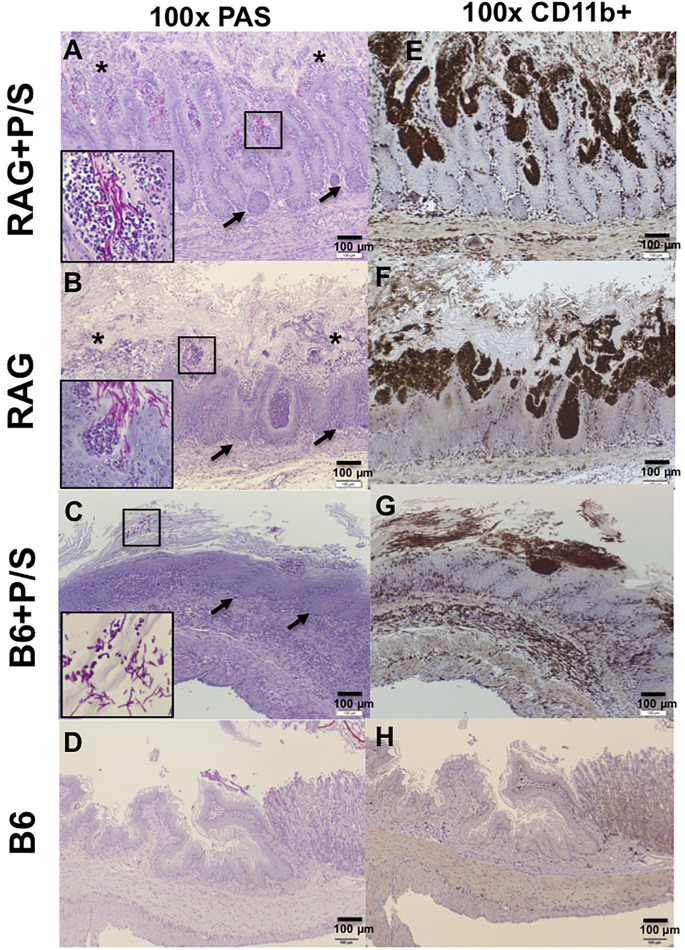
The severity of tissue damage and neutrophilic infiltration caused by *C. albicans* in Rag2γc mice were increasing after removal of the gut microbiota. Stomach tissues were harvested from Rag2γc (RAG) or B6 mice infected with 1 × 10^7^ SC5314 cells and treated with or without antibiotic (P/S) treatment 14 days after infection, rinsed with cold PBS and fixed with formalin. Tissue sections were stained with PAS **(A–D)** or subjected to anti-CD11b immunostaining **(E–H)**. Focal hyperplasia (arrows) and erosion (asterisks) of the gastric mucosa are indicated **(A,B)**. *C. albicans* hyphae in the mucosa **(A,B)** or yeast/pseudohyphae in the disrupted stratified squamous epithelium **(C)** can be clearly seen in the small image (400 × magnification) inside the boxes in the lower left corners of the images. Infiltration of CD11b^+^ inflammatory cells (brownish-black), most of which are neutrophils, accompanied *C. albicans* infection.

To understand the extent of the invasion and inflammation in *C. albicans*-colonized Rag2γc and B6 mice under the influence of antibiotics, stomach tissues collected 14 days after infection were stained with PAS and an antibody specific for CD11b, which is expressed on the surface of inflammatory cells, such as macrophages, dendritic cells, NK cells and neutrophils. PAS staining showed that fungal hyphae penetrated the mucosa in both antibiotic-treated and untreated Rag2γc mice; however, we observed a greater number of invasion sites with focal hyperplasia and erosions in the epithelial layer and a higher abundance of fungi in antibiotic-treated Rag2γc mice (Figures 5A,B). In addition, we observed that the invading fungi were surrounded by neutrophils (inside the boxed areas in Figures 5A,B). In contrast, compared with antibiotic-treated Rag2γc mice, antibiotic-treated B6 mice exhibited fewer fungal cells in the hyphal form in the mucus layer and stratified squamous epithelium, where a thick keratin layer and basal level of hyperplasia were observed (Figure 5C). Consistent with fungal penetration, CD11b^+^ inflammatory cells colocalized with fungi could be clearly seen infiltrating into the mucosa in both the antibiotic-treated and untreated Rag2γc mice (Figures 5E,F). Colocalization of CD11b^+^ inflammatory cells and fungi was also observed in the stratified squamous epithelium in antibiotic-treated B6 mice (Figure 5G). Neither fungi nor CD11b^+^ cell infiltration was detected in the untreated B6 mice (Figures 5D,H).

### Increased Levels of Inflammatory Cytokines and Lower Levels of IL-22 Were Detected in *C. albicans*-Infected Rag2γc Mice

**FIGURE 6:**
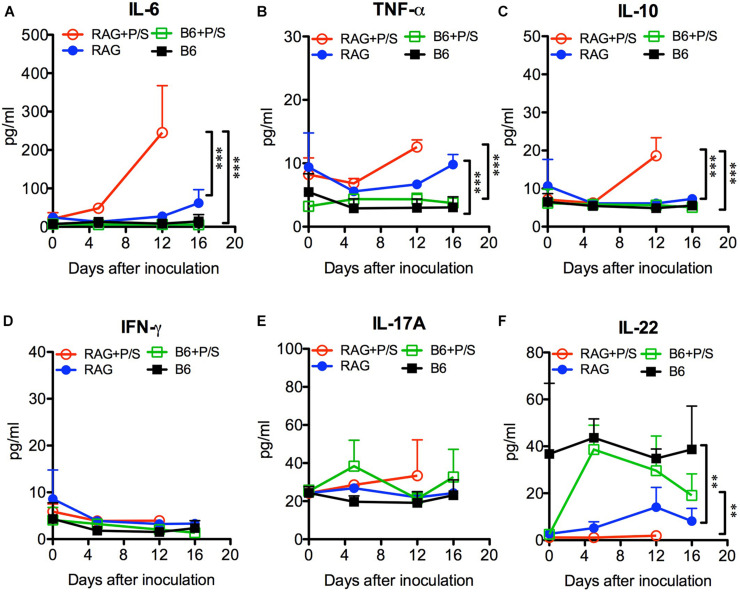
Changes in plasma cytokine levels in *C. albicans*-infected mice. Rag2γc (RAG) or B6 mice (*n* = 4) were either pretreated or not treated with antibiotics (P/S) and infected with 1 × 10^7^ SC5314 cells by oral gavage. Sera were collected for cytokine profile analysis by a Milliplex cytokine ELISA. The means and SD of the plasma levels of IL-6 **(A)**, TNF-α **(B)**, IL-10 **(C)**, IFN-γ **(D)**, IL-17A **(E)**, and IL-22 **(F)** are shown in pg/ml.

To further investigate the systemic inflammation caused by *C. albicans* infection, we measured the levels of plasma cytokines by ELISA. Sepsis-associated inflammatory cytokines, including IL-6, TNF-α, and IL-10, were significantly elevated in antibiotic-treated Rag2γc mice 12 days post infection compared to their levels in the B6 mice (*p* < 0.001; Figures 6A–C). The untreated Rag2γc mice with lower tissue fungal burdens also exhibited increased levels of plasma IL-6 and TNF-α on day 16 compared with those in untreated B6 mice. Although antibiotic-treated B6 mice had higher tissue fungal burdens, their unchanged plasma IL-6, TNF-α and IL-10 levels correlated with the lack of gastric mucosal damage upon histological findings. In addition to inflammatory cytokines, Th1 and Th17 cytokines are reported to play a role in the host defense against systemic and mucosal candidiasis; therefore, we measured the changes in plasma IFN-γ, IL-17A, and IL-22 levels. Neither Rag2γc nor B6 mice showed any increase in IFN-γ or IL-17A (Figures 6D–E). However, the basal level of plasma IL-22 was reduced in antibiotic-treated B6 mice compared to untreated B6 mice but after *C. albicans* GI infection, the plasma IL-22 level was increased to a level comparable to that in untreated B6 mice (Figure 6F). The basal level of plasma IL-22 in both antibiotic-treated and untreated Rag2γc mice was as low as the level in antibiotic-treated B6 mice. After *C. albicans* GI infection, the level of plasma IL-22 in Rag2γc mice remained significantly lower than the level as compared to that in B6 mice, although the level of IL-22 was slightly elevated in untreated Rag2γc mice 12 days after infection. This finding suggested that the level of IL-22 in the Rag2γc mice was still limited.

## Discussion

The current study demonstrated that *C. albicans*, a major fungal species causing opportunistic infections, colonized the gut and spontaneously caused disseminated candidiasis in severe combined immunodeficient adult mice without any pretreatments. This GI colonization and dissemination were shown to be dose-dependent and filamentation-required. Tissue lesions caused by invaded fungi were observed in the stomach, where a significant inflammatory response, including mucosal neutrophilia and high levels of IL-1β expression, was induced, leading to fatality within 4 weeks. Removal of gut bacteria by antibiotics accelerated the progression of the *C. albicans* invasion and mouse mortality. The significantly lower levels of IL-22 in Rag2γc mice than those in B6 mice suggest a role of IL-22 in the susceptibility to *C. albicans* invasion. Thus, this model with pathological features of candidiasis allows investigation of the mechanisms underlying *C. albicans* colonization and pathogenesis.

In humans, *C. albicans* interacts with the microbiota and host mucosal immunity to maintain its commensal colonization; however, *C. albicans* is not a natural commensal in the gut of adult mice. Reports have indicated that the existing gut bacteria, particularly anaerobic bacteria, efficiently stimulate epithelial cells to secrete antimicrobial peptides and maintain cell integrity via the IL-17/IL-22 signaling pathway to block *C. albicans* adhesion in immunocompetent mice (Zindl et al., 2013; Conti and Gaffen, 2015; Fan et al., 2015). Consistent with these reports, we found that *C. albicans* could colonize B6 mice only after the removal of gut bacteria by antibiotic treatment. However, the unique novelty of the current immunodeficient mouse model is that antibiotic pretreatment was not required for *C. albicans* colonization. This natural *C. albicans* colonization in Rag2γc mice might be due to the differences in the gut microbiota; however, the abundance of total gut bacteria was similar between the Rag2γc and B6 mice (Figure 4A) and the composition of gut bacteria, including two major anaerobic phyla (*Bacteroidetes* and *Firmicutes*) and two major genera (*Bacteroides* and *Blautia*) displaying the inhibition to *Candida* colonization (Fan et al., 2015), showed no significant difference between two strains of mice in a preliminary study (Supplementary Figure 1). Instead, the downstream IL-22 production stimulated by the gut microbiota (Satoh-Takayama et al., 2008) might be the key contributor. IL-22 is important for antimicrobial peptide production and gut homeostasis (Hooper et al., 2012; Kamada et al., 2013). Indeed, we observed that IL-22 levels were negatively associated with antibiotic treatment in B6 mice; however, the much lower level of plasma IL-22 in Rag2γc mice regardless of antibiotic treatment (Figure 6F) suggested that the lower IL-22 response to the gut bacteria in the Rag2γc mice might contribute to the breakdown of colonization resistance. This finding is not surprising because IL-22 knockout mice also exhibit a similar phenotype; in these mice, *C. albicans* can adhere or penetrate into the stomach mucosa, but subsequent dissemination does not occur (De Luca et al., 2010), possibly due to protective Th1 and Th17 responses to systemic infection (Richardson and Moyes, 2015). In Rag2γc mice, the deficits in both Th1 and Th17 cells as well as the main IL-22 producing lymphocytes, including T, NK, and innate lymphoid cells (Van Maele et al., 2014), allow *C. albicans* to disseminate from the GI tract. In addition to lymphoid cells, myeloid cells such as neutrophils have also been documented to produce IL-22 and contribute to antimicrobial peptide production (Zindl et al., 2013). Given that a slightly elevated level of IL-22 in untreated Rag2γc mice after *C. albicans* infection (Figure 6F), the possibility that the IL-22 producing myeloid cells play a role in the *C. albicans* infection cannot be ruled out, particularly in alymphoid Rag2γc mice.

In spite of the lower IL-22, Rag2γc mice still efficiently prevented low dose *C. albicans* infection. One explanation is that gut microbiota in Rag2γc mice inhibited low dose *C. albicans* colonization by directly competing for limited resources and secreting suppressive metabolites (Wagner and Johnson, 2012). Therefore, dysbiosis of the gut microbiota caused by antibiotic treatment (Figure 4A and Supplementary Figure 1D) has a deleterious effect by increasing the *C. albicans* colonization to enhance the severity of fungal infections in the Rag2γc mice. It is particularly interesting to note that fungal burden varied between different compartments of the GI tract in the Rag2γc mice. It has been reported that the composition of the microbiota varies between different compartments of the GI tract (Kamada et al., 2013), thus resulting in the different inhibitory effects on fungal colonization. For example, fungal burden in the colon of untreated Rag2γc mice was lower than that in treated mice, while the fungal burden in the stomach was similar between antibiotic-treated and untreated Rag2γc mice (Figure 4E). It suggests that bacteria residing in the colon inhibited *C. albicans* colonization more efficiently than those residing in the stomach and thus might account for the higher fungal burden in the stomach of the untreated Rag2γc mice (Figures 1D, 4E). Although *C. albicans* predominantly colonized the colon in other antibiotic-treated mouse models (Wiesner et al., 2001), the 1000 times higher fecal fungal counts in the high dose SC5314-infected Rag2γc mice than those in moderate dose groups (Figure 1A) correlated to the fungal burden in the stomach but not that in the colon (about 1000 and 10 times higher in high dose group for the stomach and colon, respectively; Figure 1D) suggests that the stomach was the dominant colonization site in this model.

In addition to the inhibition by gut microbiota, neutrophil can also provide protection against *Candida* invasion by killing engulfed fungi through oxidative and non-oxidative mechanisms, or by trapping extracellular fungi by neutrophil extracellular traps (NETs) (Netea et al., 2015; Davidson et al., 2018). If the hyphal form grows excessively, epithelial cells can produce inflammatory mediators to recruit innate immune cells to clear fungal infections (Naglik et al., 2017). Our current study supported this idea by demonstrating that the colocalization of CD11b^+^ inflammatory cells and fungi in the stomach of both Rag2γc and B6 mice. In addition, that more neutrophils were recruited to *C. albicans* infection sites enriched with the hyphal form in Rag2γc mice compared to those with less hyphal form in B6 mice (Figures 5A vs. 5D) suggested the efficient recruitment of neutrophils in Rag2γc mice. However, in the absence of lymphoid cells, Rag2γc mice still failed to protect from the high dose *C. albicans* infection.

Moreover, beside the microbiota and host immunity, the virulence of *C. albicans* also influences its colonization. Previous reports have demonstrated that the wild-type strain and the filamentation-defective HLC54 mutant showed no difference in intestinal colonization in an antibiotic-pretreated mouse model (Henry-Stanley et al., 2005); however, our data clearly showed that in the presence of the gut microbiota, HLC54 failed to persistently colonize the gut in Rag2γc mice and did not subsequently disseminate (Figure 2). These findings are consistent with the recent report that the virulent wild-type *C. albicans* persisted in mice gut if an intact microbiota was present but gradually lost its filamentation ability if the gut microbiota were removed by antibiotic treatment (Tso et al., 2018). In addition to colonization, hyphal formation is also essential for *C. albicans* pathogenesis (Saville et al., 2003). Most virulence genes are hyphae-associated genes and are expressed after the transition from the yeast to the hyphal form. *C. albicans* hyphae can efficiently break mucosal barriers via different mechanisms, including activation of NLRP3-dependent inflammation by candidalysin to cause epithelial damage (Allert et al., 2018). In SC5314-infected Rag2γc mice, the increase in the expression of IL-1β (Figure 4F) also suggested gut epithelial damage through NLRP3-dependent inflammation. In addition, the coexistence of infiltrating neutrophils and *C. albicans* hyphae in this damaged epithelium implicated that both may be involved in the tissue inflammation. In a model of *Pseudomonas aeruginosa* pneumonia, the levels of IL-22 were reported to negatively correlate to tissue damage with an increase in neutrophil accumulation in lungs (Broquet et al., 2017). Considering the deficits in IL-22 in Rag2γc mice and the accumulation of neutrophils in the damaged epithelium, it is reasonable to speculate that activated neutrophils contributed to the severe inflammation observed. As mice became moribund, the severity of tissue injury increased by penetration of hyphae into the muscular layer (Figure 3I) and the plasma levels of IL-6, TNF-α, and IL-10 were also elevated (Figure 6). Notably, the possibility that the increases in these septic-associated inflammatory cytokines was caused by bacterial leakage from the gut lumen cannot be ruled out, because mixed candidemia and bacteremia were found in approximately 18% of patients with candidemia in a retrospective cohort study (Bouza et al., 2013). Another clinical sign for systemic inflammation might be the body weight loss. In contrast to B6 mice, only Rag2γc mice infected with high dose of *C. albicans* SC5314 displayed a significant body weight decrease. Interestingly, the Rag2γc mice infected with moderate or low dose of SC5314 showed the body weight gain, compared to the unchanged body weight in untreated B6 mice (Figure 1B). The reasons for this unexpected body weight change remain unclear; however, the similar body weight increase in antibiotic-treated B6 mice after infection suggested that the gut microbiota changes caused by *C. albicans* colonization might be involved in the body weight gain observed in the Rag2γc mice infected with moderate or low dose of *C. albicans*.

The fungal burden in the liver and/or kidney is a hallmark for *C. albicans* dissemination but was hardly seen in the Rag2γc mice 12 days after infection (Figure 1D) except occasionally in the antibiotic-treated Rag2γc mice (Figure 4E). It might be too early for *C. albicans* translocation; therefore, the results of moribund mice sacrificed 15–22 days after infection were used for analysis. Evidence of detectable fungi in the liver and/or kidneys in 58% of moribund mice (Figure 2D) suggested the dissemination of gut-originated *C. albicans*. This percentage might be underestimated because the infected mice might be sacrificed prior to the *C. albicans* dissemination (humane endpoints were performed within 24 h when body weight loss was ≥20%) or die suddenly to lose the tissue harvest. However, the fungal burden in liver and/or kidneys was not as high as that seen in lethal intravenous infection models (Bar et al., 2014). Alternatively, the severe gastritis with high levels of sepsis-associated inflammatory cytokines in the circulation, not liver and/or kidney failure, was probably the major cause of death in *C. albicans*-infected Rag2γc mice.

As a corollary, spontaneously developed dissemination from endogenous *C. albicans* has also been reported in germ-free immunodeficient *bg/bg* × *nu/nu* mice (Cantorna and Balish, 1990), which have defects in T, B and NK cells and have abnormally functioning in granulocyte chemotaxis (Gallin et al., 1974). In this germ-free mouse model, *Candida albicans* colonized the gut, resulting in mucosal infection in the stomach, and caused progressive systemic infection and mortality 12–16 weeks after colonization. Our data in the Rag2γc mouse model partially correlate with the findings in germ-free *bg/bg* × *nu/nu* mouse results, but also further demonstrate disseminated candidiasis within 4 weeks from colonization, transition to translocation in the presence of gut microbiota.

Our studies revealed that *C. albicans* was able to colonize Rag2γc mice in the presence of the gut microbiota and to penetrate the gastric mucosa, resulting in severe inflammation and killing all mice within 4 weeks. Although host immunity was severely debilitated in this mouse model, the gut microbiota and mucosal barriers remained intact, which allowed us to better dissect the interaction between the fungi and the microbiota, examine fungal virulence during translocation through the alimentary tracts, and evaluate antifungal therapy. In addition, this unique mouse model also revealed the feasibility of studying the synergism and antagonism between gut bacteria and *C. albicans* in GI colonization, transition and translocation.

## Materials and Methods

### Ethics Statement

The immunocompetent female C57BL/6 (B6) mice (The National Laboratory Animal Center, Taipei, Taiwan) and immunocompromised randomized male and female Rag2^–/–^IL2γc^–/–^ (Rag2γc) mice (Taconic Farm, Bar Harbor, ME, United States), which were bred locally under the agreement, were used in this study and maintained in the animal facility of the National Health Research Institutes, Taiwan. The protocol was approved by the Animal Committee of the National Health Research Institutes (protocol no: NHRI-IACUC-103013-A) and performed according to their guidelines.

### *Candida albicans* Strain and Culture Conditions

The wild-type *C. albicans* strain SC5314 and its filamentation-defective mutant HLC54 (*efg1/efg1 cph1/cph1*) (Lo et al., 1997) were used in this study. The strains were stored in vials at −70°C and plated on Sabouraud dextrose agar (SDA) to grow overnight. Refreshed colonies were continually grown in yeast extract-peptone-dextrose (YPD) broth at 37°C overnight, washed and resuspended in phosphate-buffered saline (PBS). The *C. albicans* concentration was determined using a hemocytometer.

### Murine Model of *C. albicans* GI Colonization and Subsequent Dissemination

Mice were housed individually in ventilated cages in a specific-pathogen-free environment and supplied with sterile bedding, water and food. For infection, 6- to 8-week-old mice were moved to a separate room 5 days before the experiments, and were then orally injected with *Candida* by gavage (20G-50, Natsume Seisakusho Co., Ltd., Tokyo, Japan). Body weight was monitored every 2–3 days until the mice became moribund (defined as a decrease of over 20% of the initial body weight or inability of a mouse to feed itself). Stool was collected from individual mice every 2–3 days, weighed and homogenized in 0.5 ml of PBS. Then, 200 μl of serial 10-fold dilutions of the homogenates were plated on YPD plates to evaluate GI colonization. For fungal burden determination, tissues (the stomach, small intestine, ileum, colon, liver, and kidney) were harvested, rinsed with PBS, weighed and homogenized in 0.5 ml of PBS. Then 200 μl of the homogenates were plated on YPD plates for growth and subsequent counting. In the antibiotic-treatment experiment (Figure 4), 1500 U/ml of streptomycin and 2000 U/ml of penicillin were added to the drinking water to remove the gut bacteria beginning 5 days before *C. albicans* inoculation and continuing until the end of the experiment.

### Tissue Staining

Tissue specimens were obtained from *C. albicans*-infected mice, rinsed with PBS and fixed with formalin. Tissues were embedded in paraffin, sectioned and subjected to either hematoxylin and eosin (H-E) or periodic acid-Schiff (PAS) staining. CD11b^+^ cells were identified using a rat monoclonal antibody specific for mouse CD11b (Dako, Glostrup, Denmark) and a horseradish peroxidase (HRP)-conjugated rabbit anti-rat IgG secondary antibody (Dako). Color was then developed with diaminobenzidine (DAB).

### Cytokine ELISAs

Cytokine profiling was conducted using a Milliplex multiplex assay (Merck, Billerica, MA, United States). In brief, 25 μl of serum samples for each round were used in accordance with manufacturer’s directions. The serially diluted specimens and standards were assayed in a Luminex 200 system and analyzed by Milliplex Analyte software version 5.1.

### Quantitative PCR or RT-PCR

Stool samples from individual mice were weighed and homogenized in 0.5 ml of PBS. Fecal DNA was purified using the MPbio DNA/RNA isolation kit (MP Biomedicals, Solon, OH, United States) based on the manufacturer’s directions. The copy number of the conserved bacterial 16S rRNA gene was determined by quantitative PCR with the following primer and probe sets: forward, 5′-GGTGAATACGTTCCCGG-3′; reverse, 5′-TACGGCTACCTTGTTACGACTT-3′; probe, FAM-CTTGTACACACCGCCCGTC-TAMRA. For quantitative RT-PCR, the total RNA was isolated from tissue homogenates using TRIzol (Invitrogen, Carlsbad, CA, United States) and an RNA clearance kit (Qiagen, Hilden Germany), and was then reverse transcribed to cDNA using Superscript III (Invitrogen) and stored at −80°C until use. The levels of IL-1β cDNA were determined by quantitative PCR and normalized to the cDNA levels of the housekeeping gene GAPDH with the following TaqMan primer and probe sets: forward, 5′-AACCTGCTGGTGTGTGACGTTC-3′, reverse, 5′-CAGCACGAGGCTTTTTTGTTGT-3′, and probe, FAM-TTAGACAGCTGCACTACAGGCTCCGAGATG-TAMRA for IL-1β; forward, 5′-CAATGTGTCCGTCGTGGATCT-3′, reverse, 5′-GTCCTCAGTGTAGCCCAAGATG-3′, and probe FAM-CGTGCCGCCTGGAGAAACCTGCC-TAMRA for GAPDH.

### Gut Microbiota Analysis

Stool from individual mice were weighed and homogenized in 0.5 ml of PBS. Fecal DNA was purified by the MPbio DNA/RNA isolation kit based on the direction of manufacture. The bacteria 16S rRNA gene was determined by illumine sequencing. In brief, the bacterial 16S rRNA genes were amplified by KAPA High-Fidelity PCR Master Mix (KAPA BIOSYSTEMS) using the specific primers targeting the V3 and V4 hypervariable regions (341F-CCTACGGGNGGCWGCAG and 805R- GACTACHVGGGTATCTAATCC) with the barcodes. The PCR product was purified with QIAquick gel extraction kit (QIAGEN). Sequencing libraries were generated using TruSeq Nano DNA Library Prep Kit (Illumina, United States) following manufacturer’s recommendations and index codes were added. The library was sequenced on an Illumina MiSeq platform to generate 300 bp paired-end reads. The operational taxonomic units (OTUs) generated from quality-filtered and non-chimeric reads were analyzed with SILVA 132 OUT collection for bacterial OUT taxonomy assignment.

### Statistical Analyses

All statistical analyses were performed with 2-way ANOVAs with the Bonferroni posttest (GraphPad Prism), unless otherwise specified. Differences with a *p*-value of less than 0.05 were considered statistically significant.

## Summary

The mechanism of fungal translocation from the gut mucosa to the bloodstream is poorly understood, partially because relevant animal models are lacking. In contrast to humans, adult mice are resistant to *C. albicans* colonization unless gut bacteria are removed through the use of broad-spectrum antibiotics. In addition, immunosuppressive and/or chemotherapeutic agents are also required to induce mucosal barrier damage to facilitate fungal invasion. These pretreatments interfere with studies of *C. albicans* translocation and interaction with the gut microbiota. We used severe combined immunodeficient mice with defects in T, B, and NK cell development to demonstrate the natural colonization and spontaneous dissemination of *C. albicans*. Our studies revealed that *C. albicans* was able to colonize Rag2γc mice in the presence of the gut microbiota and could penetrate the gastric mucosa, where a significant inflammatory response, including mucosal neutrophilia, was induced by the invading fungi, leading to mortality within 4 weeks. Thus, this model with pathological features of candidiasis offers an opportunity to investigate the mechanisms of *C. albicans* colonization and pathogenesis.

## Data Availability Statement

The original contributions presented in the study are publicly available. This data can be found here: https://data.mendeley.com/datasets/4gfs7mmm2c/2.

## Ethics Statement

The animal study was reviewed and approved by the Animal Committee of the National Health Research Institutes (protocol no: NHRI-IACUC-103013-A).

## Author Contributions

C-HP, H-JL, and Y-CC: experimental design and manuscript writing. C-HP, J-WH, and D-WL: data analysis. J-YY, Y-JH, J-WH, D-WL, T-HL, and S-HW: experiment conduct. All authors contributed to the article and approved the submitted version.

## Conflict of Interest

The authors declare that the research was conducted in the absence of any commercial or financial relationships that could be construed as a potential conflict of interest.
