# Supplementary material for: Candida albicans Colonizes and Disseminates to the Gastrointestinal Tract in the Presence of the Microbiota in a Severe Combined Immunodeficient Mouse Model
Source: Front Microbiol. 2021 Jan 8;11:619878. doi: 10.3389/fmicb.2020.619878 (PMC7819875; doi:10.3389/fmicb.2020.619878)
Supplement: Supplementary file 1 [file Image_1.pdf]

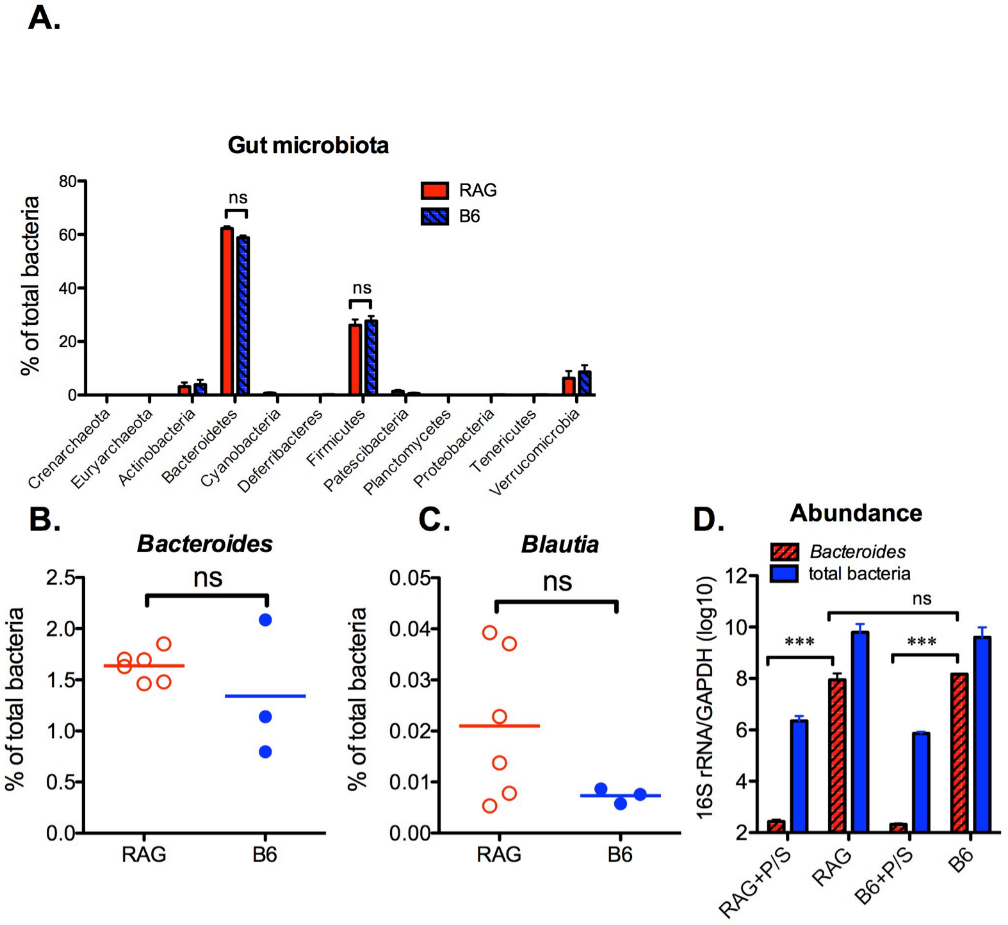

Fig S1. The composition and abundance of microbiota showed no significant difference between Rag2γc and C57BL/6 mice. Fecal bacterial DNA from uninfected Rag2γc (RAG; n=6) and C57BL/6 (B6; n=3) mice with or without antibiotic treatment (P/S) were isolated by MPbio DNA isolation kit and analyzed the composition of microbiota by 16S rRNA gene sequencing. The composition of bacteria in phylum level (A) and the relative abundance of genera *Bacteroides* (B) and *Blautia* (C) in total bacteria are showed. The abundance of total bacteria and genus *Bacteroides* was measuring by quantitative PCR and the results were normalized to GAPDH and presented by the mean and SD (D). For statistical analysis, an unpaired t-test with Welch's correction (B and C) and 2-way ANOVA (D) are used. The raw data was available in https://data.mendeley.com/datasets/4gfs7mmm2c/2
